# Performance Characteristics of the Cepheid Xpert MTB/RIF Test in a Tuberculosis Prevalence Survey

**DOI:** 10.1371/journal.pone.0043307

**Published:** 2012-08-15

**Authors:** Susan E. Dorman, Violet N. Chihota, James J. Lewis, Maunank Shah, David Clark, Alison D. Grant, Gavin J. Churchyard, Katherine L. Fielding

**Affiliations:** 1 Johns Hopkins University School of Medicine, Baltimore, Maryland, United States of America; 2 Aurum Institute for Health Research, Johannesburg, South Africa; 3 London School of Hygiene and Tropical Medicine, London, United Kingdom; 4 School of Public Health, University of Witwatersrand, Johannesburg, South Africa; Institute of Infectious Diseases and Molecular Medicine, South Africa

## Abstract

**Background:**

Xpert MTB/RIF (“Xpert”) is a molecular test for detection of *Mycobacterium tuberculosis* (MTB) in sputum. Performance characteristics have been established for its use during passive tuberculosis (TB) case detection in symptomatic TB suspects, but Xpert performance has not been assessed in other settings. Objectives were to determine Xpert performance and costs in the context of a TB prevalence survey.

**Methodology/Principal Findings:**

This was a diagnostic sub-study of a TB prevalence survey conducted in gold mining companies in South Africa. Sputa (one per participant) were tested using smear microscopy, liquid culture (reference comparator), and Xpert. Costs were collected using an ingredients approach and analyzed using a public health program perspective. 6893 participants provided a sputum specimen. 187/6893 (2.7%) were positive for MTB in culture, 144/6893 (2.1%) were positive for MTB by Xpert, and 91/6893 (1.3%) were positive for acid fast bacilli by microsocopy. Sensitivity, specificity, positive predictive value, and negative predictive value for detection of MTB by Xpert were 62.6% (95% confidence interval [CI] 55.2, 69.5), 99.6% (99.4, 99.7), 81.3% (73.9, 87.3), and 98.9 (98.6, 98.8); agreement between Xpert and culture was 98.5% (98.2, 98.8). Sensitivity of microscopy was 17.6% (12.5, 23.9). When individuals with a history of TB treatment were excluded from the analysis, Xpert specificity was 99.8 (99.7, 99.9) and PPV was 90.6 (83.3, 95.4) for detection of MTB. For the testing scenario of 7000 specimens with 2.7% of specimens culture positive for MTB, costs were $165,690 for Xpert and $115,360 for the package of microscopy plus culture.

**Conclusion:**

In the context of a TB prevalence survey, the Xpert diagnostic yield was substantially higher than that of microscopy yet lower than that of liquid culture. Xpert may be useful as a sole test for TB case detection in prevalence surveys, particularly in settings lacking capacity for liquid culture.

## Introduction

Tuberculosis (TB) prevalence surveys have been used to assess the performance of TB control programs, provide information for strategic planning, and assess trends of disease burden over time [1], [2], [3], [4]. TB prevalence surveys are increasingly being used to measure the impact of large-scale interventions such as isoniazid preventive therapy and enhanced TB case finding [5], [6], [7], [8]. Historically in prevalence surveys, pulmonary TB case detection has been based on microbiological evaluation using sputum smear microscopy; symptom assessment and chest radiographs are sometimes used as initial screens in order to identify individuals who subsequently undergo microbiological evaluations in order to maximize efficiency and minimize laboratory costs [2]. Few prevalence surveys have included mycobacterial culture, which is considered the gold standard for individual patient-based TB diagnosis but has substantial infrastructure and labor requirements and is prone to contamination [4]. Sputum smear-based surveys are problematic due to the low sensitivity of smear especially in HIV-related TB [9]–[11] and the inability of smear to provide information about drug resistance, an understanding of which is critical for TB control. Mycobacterial culture of sputum is complex and relatively expensive at the laboratory level, and depends on sputum transport conditions that retain *M. tuberculosis* (MTB) viability without promoting growth of contaminating organisms.

Xpert MTB/RIF (Cepheid, Sunnyvale, CA) is an integrated specimen processing and nucleic acid amplification-based test for detection of MTB and rifampin resistance-conferring mutations directly from sputum [12]. As used for passive TB case detection in respiratory symptomatic adults in the context of a clinical validation trial and a large multisite demonstration study in which overall TB prevalences among tested participants were 49% and 25%, respectively, Xpert MTB/RIF (“Xpert”) had sensitivities of approximately 98% for smear-positive/culture positive TB and 72.5% to 76.9% for smear-negative/culture-positive disease, with specificity of approximately 99% [13], [14]. It is worth pointing out that these studies did not include anyone on TB treatment.

Several Xpert characteristics besides relatively high sensitivity make it attractive for use in population-based surveys. First, unlike culture-based tests, Xpert does not require viable bacilli, thereby potentially making specimen storage and transport conditions less demanding. Second, the risk of specimen-to-specimen cross contamination with MTB appears to be very low for the closed cartridge Xpert test, based on data from a research laboratory [15]. Third, the automated performance of almost all Xpert testing steps within the closed cartridge manufactured using quality controls would be expected to lessen lab-to-lab variability, which can be problematic in large prevalence surveys using culture. Fourth, Xpert provides information about rifampicin resistance, which is highly associated with multidrug-resistance and poor treatment outcomes using conventional TB treatment regimens [13], [14]. However, the latter potential attribute must be tempered by reports of false-positive rifampicin resistance results by Xpert [14], [16], [17], [18]. The total Xpert run time is about 2 hours, substantially less than that for cultures. Finally, for Xpert procedures the laboratory biosafety risks are limited and therefore extensive biocontainment infrastructure is not required [13], [19].

We incorporated Xpert testing into a TB prevalence survey in which MGIT culture and smear microscopy were already being performed on all sputa. The overall study goal was to determine the performance of Xpert when used for TB case ascertainment in the context of a population-based TB prevalence survey.

## Methods

### Design and Setting

This was a diagnostic sub-study of a cross-sectional TB prevalence survey conducted in gold mining companies in South Africa. The parent prevalence survey was conducted to measure a secondary endpoint of the ‘Thibela TB’ study, a cluster randomized trial of the impact of community-wide TB preventive therapy on the burden of TB in a setting of high HIV prevalence [5]. This diagnostic sub-study was conducted in 12 of the 15 clusters (mine shafts) participating in the Thibela TB study.

### Participants

Participants were recruited based on a consecutive sample of employees attending occupational health services for their annual fitness examination from April 2010 to May 2011. Demographic information and targeted information about current and previous TB treatment and HIV were obtained from participants through interviews with study staff. HIV testing was not a component of the ‘Thibela TB’ study or this diagnostic sub-study. Participants were provided with information about how to expectorate a deep sputum specimen. One spontaneously expectorated respiratory specimen was collected from each participant; any specimen produced was tested. Sputa were briefly stored at 4°C, and transported to the study laboratory within 24 hours of collection.

### Laboratory Methods

All tests were conducted by a single reference laboratory with quality-assured smear microscopy and culture. Sputa were decontaminated using N-acetyl-L-cysteine-NaOH, with final [NaOH] of 1% [20]. After centrifugation, the pellet was suspended in approximately 1.5 ml of phosphate buffer pH6.8. A Ziehl-Neelsen-stained smear of the decontaminated sediment was examined by light microscopy and graded according to WHO criteria [21]. A 0.5 ml portion of the sediment was inoculated into the BACTEC MGIT 960 system (BD Diagnostic Systems, Sparks, MD) using Mycobacterial Growth Indicator Tubes (MGITs, BD Diagnostic Systems). Positive cultures were confirmed as mycobacteria using Ziehl-Neelsen staining, and as MTB using an anti MPB64 monoclonal antibody assay (Capilia TB-Neo, TAUNS Laboratories, Numazu, Japan) test. Indirect phenotypic drug susceptibility testing was performed using the MGIT SIRE system (BD Diagnostic Systems) only for isolates for which the sputum had been rifampicin resistant by Xpert.

Xpert MTB/RIF tests were conducted and interpreted according to the manufacturer’s recommendations. Xpert MTB/RIF kit sample reagent was added in a 3∶1 (volume:volume) ratio to 0.5 ml of the decontaminated sputum pellet, incubated at room temperature for 15 minutes, and 2 ml of this mixture was added to an Xpert MTB/RIF cartridge. A 4-module GeneXpert (Cepheid) instrument with automated readout was used. An Xpert test was considered “failed” if a definitive reading of MTB detected or MTB not detected could not be obtained on two test attempts from the same decontaminated sputum pellet, or on one attempt if there was insufficient remaining specimen volume for a second attempt.

### Statistical Analysis

Laboratory data were single entered and source verified into a database and discrepancies were checked against the source data. Demographic data were captured directly onto a database at the participant interview. Diagnostic yield was defined as the (number of positive tests/total # tests)×100. Xpert sensitivity, specificity, and predictive values were calculated using MGIT culture results as the reference standard; specimens were excluded from these analyses if the MGIT result was “contaminated” (since either the presence or absence of MTB could not be established) or if the Xpert result was “failed”. A culture was considered positive if *M. tuberculosis* (identified to the species level) was detected; cultures with no growth or with growth of a mycobacterium other than *M. tuberculosis* were considered negative for analysis purposes. Comparisons of proportions were performed using McNemar’s chi-square and Fisher’s exact tests for paired and unpaired data, respectively, and 95% confidence intervals (CIs) were calculated using the binomial exact method. Odds ratios using logistic regression were used to assess the association between previous and current TB with “false positive” detection of MTB by Xpert, where “false positive” by Xpert was defined as a test result of positive for MTB by Xpert yet negative for MTB by MGIT.

### Economic Evaluation

Laboratory costs associated with conducting Xpert testing were compared with those associated with using a package of smear microscopy with MGIT culture along with organism identification and MGIT-based phenotypic drug susceptibility testing (DST) using the SIRE system (Becton Dickinson). Costs were collected at the National Health Laboratory Service (NHLS) reference TB laboratory in Johannesburg, South Africa. An “ingredients” approach was used that involved multiplying the quantity of inputs used by their unit prices. Costs were considered from the time of specimen receipt at the laboratory through reporting of test results. Costs associated with sputum collection and transportation were not included. The amount of staff time, consumable supplies, and equipment quantities utilized for each test were determined through direct observation of testing procedures, and included costs associated with laboratory quality control procedures. Overhead laboratory costs included indirect labor costs, office and laboratory supplies, furniture, and physical infrastructure (building, utilities, maintenance, medical waste disposal). Overhead costs were allocated for each test system based on the volume of testing and physical infrastructure utilized by each diagnostic system. Costs were gathered using detailed laboratory budget records. Capital item costs and maintenance costs were estimated from manufacturer or distributor quotations and NHLS laboratory invoices; capital item costs were annualized over items’ useful lifespans. Building rental costs and utilities were estimated based on 2011 commercial real estate pricing in Johannesburg. Costs for Xpert instrument and reagent kits reflected the reduced pricing structure negotiated by the Foundation for Innovative New Diagnostics for the South African public sector. For the base-case we assumed that the lab was running at 100% capacity (8–12 hours/day for five days/week). Costs for microscopy were based on Ziehl-Neelsen staining and light microscopy. Costs for staff time during the ∼120 minute Xpert running time were not included. A currency conversion rate of 6.978 South African rand per 1.00 U.S. dollar was used – this was the average conversion rate for 2011 at the time of the final data analysis.

### Ethics

This study was approved by ethics boards of the University of the KwaZulu Natal, the London School of Hygiene and Tropical Medicine, and the Johns Hopkins University School of Medicine. All participants provided written or witnessed verbal informed consent. Verbal informed consent was allowed in order to not exclude illiterate participants from this minimal risk study; the verbal consent process was witnessed by an impartial witness. The verbal consent process was explicitly approved by the ethics boards listed above. Principals expressed in the Declaration of Helsinki were followed during the conduct of this research.

## Results

During the study period 7346 individuals were enrolled. Fifteen were unable or unwilling to provide a sputum specimen, resulting in 7331 collected sputa. Among those sputa were 438 for which one or more of the three protocol-specified tests (smear microscopy, MGIT culture, Xpert) were not performed due to either administrative error or lack of Xpert cartridges. Therefore the main analysis set was comprised of 6893 sputa from 6893 consecutive participants. Participant characteristics are shown in Table 1. Almost all were black males, reflecting the composition of the gold mining workforce. Of those who reported ever having had an HIV test, 14.5% (602/4156) reported their HIV status as positive. At the time of the study interview and sputum collection, 60/6893 individuals (0.9%) reported that they were currently on treatment for active TB (‘current TB treatment’), 833/6893 (12.1%) reported a prior history of treatment for active TB (‘prior TB treatment’), and 5994 (87.0%) reported no current or prior TB treatment.

**Table 1:** Baseline characteristics of 6893 study participants.

Characteristic		N	%
Male sex		6469	93.9
Race	Black/African	6758	98.0
	White/European	107	1.6
	Mixed race	28	0.4
Median age (IQR) in years		43 (34, 49)	
HIV status by participant verbal report	‘Positive’	602	8.7
	‘Negative’	3455	50.1
	‘Don’t know’	99	1.4
	Missing^a^	2737	39.7
Symptoms	Cough ≤2 weeks	91	1.3
	Cough >2 weeks	138	2.0
	Night sweats	226	3.3
	Weight loss	106	1.5
Current and prior TB treatment	Current TB treatment^b^	60	0.9
	Prior TB treatment^c^	833	12.1
	No history of current or prior TB treatment	5994	87.0
	Unknown	6	0.09

Abbreviation: IQR, interquartile range.

a of these, 2613 reported never having had an HIV test, 122 had an HIV test but were not willing to disclose results, and 2 had missing data.

b on treatment for active TB when the study sputum specimen was obtained.

c prior treatment for active TB but not on TB treatment when the study sputum specimen was obtained.

**Figure 1:**
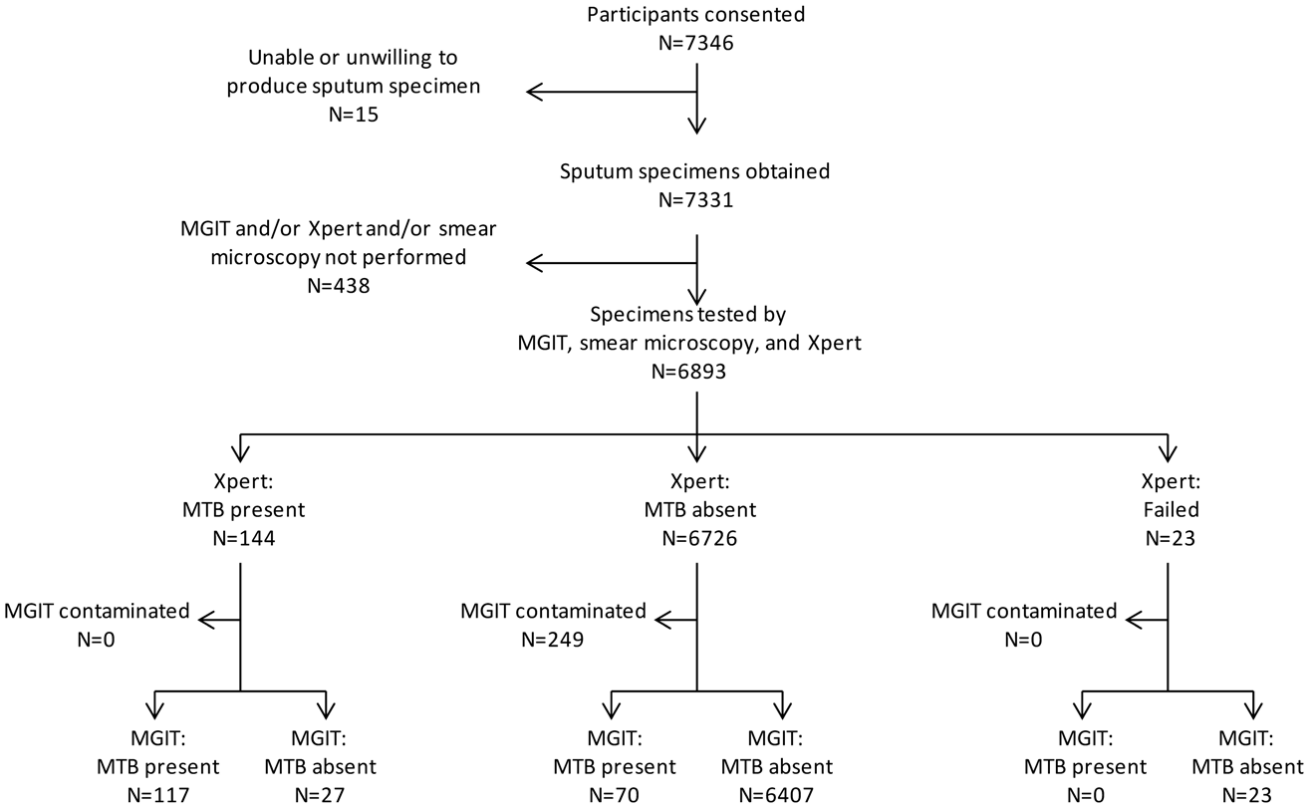
Flow diagram of Xpert and MGIT testing results.

Figure 1 and Table 2 show the results of MGIT and Xpert tests for all 6893 specimens. By MGIT testing 187 (2.7%) were positive for MTB, 389 (5.6%) were positive only for growth of non-tuberculosis mycobacteria, 249 (3.6%) were contaminated, and 6068 (88.0%) were negative for any growth. By Xpert testing, 144 (2.1%) were positive for MTB, 6726 (97.6%) were negative for MTB, and 23 (0.3%) failed to yield a result. Xpert quantitation results for the 144 sputa in which that test detected *M. tuberculosis* were “high” for 6 (4.2%), “medium” for 10 (6.9%), “low” for 45 (31.3%), and “very low” for 83 (57.6%). The MTB diagnostic yield of MGIT (187/6893; 2.7%) was higher than that for Xpert (144/6893, 2.1%; p = 0.017) and either of these methods had a higher yield than smear microscopy (91/6893; 1.3%; p = 0.001 vs. Xpert yield).

**Table 2:** Results for MGIT culture and Xpert MTB/RIF for 6893 sputum specimens.

		MGIT					
		MTB	NTM	Negative	Contam-inated	Total	
**Xpert**	**MTB**	117	2	25	0	144	
	**Negative**	70	383	6024	249	6726	
	**Failed**	0	4	19	0	23*	
	**Total**	187	389	6068	249*	6893	

* p<0.001 for comparison of proportions of contaminated MGIT cultures (249/6893 [3.6%]) vs. invalid Xpert tests (23/6893 [0.3%]).

Table 3 shows Xpert performance characteristics overall and stratified by smear microscopy status, using MGIT as the reference comparator. Overall, 6621 sputa had an interpretable result for MGIT culture and Xpert; excluded from this analysis were 249 sputa with a MGIT final result of “contaminated” and 23 sputa with an Xpert final result of “failed”. Xpert sensitivity was 62.6% (95% CI 55.2, 69.5), specificity 99.6% (99.4, 99.7), positive predictive value (PPV) 81.3% (73.9, 87.3), negative predictive value (NPV) 98.9% (98.6, 99.2), and agreement 98.5% (98.2, 98.8). Xpert sensitivity and PPV were higher for smear-positive specimens than for smear-negative specimens (sensitivity 32/33 [97.0%] for smear-positives vs. 85/154 [55.2%] for smear negatives, p<0.001; PPV 32/33 [97.0%] for smear-positives vs. 85/111 [76.6%] for smear-negatives, p = 0.01). Specificity, NPV, and agreement were similar by smear status. For detection of MTB, the sensitivity of smear microscopy was 33/187 (17.6%; 12.5, 23.9) vs. Xpert sensitivity of 62.6%; p≤0.001).

**Table 3:** Performance characteristics of the Xpert MTB/RIF test overall, and stratified by smear microscopy status, using MGIT culture as the reference standard.

		Overall	Smear-positive	Smear-negative
		Total N = 6621^a^	Total N = 87^b^	Total N = 6534
**Xpert sensitivity**	N/N	117/187	32/33	85/154
	% (95% CI)	62.6 (55.2, 69.5)	97.0 (84.2, 99.9)	55.2 (47.0, 63.2)
**Xpert specificity**	N/N	6407/6434	53/54^c^	6354/6380
	% (95% CI)	99.6 (99.4, 99.7)	98.2 (90.1, 100.0)	99.6 (99.4, 99.7)
**Xpert PPV**	N/N	117/144	32/33	85/111
	% (95% CI)	81.3 (73.9, 87.3)	97.0 (84.2, 99.9)	76.6 (67.6, 84.1)
**Xpert NPV**	N/N	6407/6477	53/54	6354/6423
	% (95% CI)	98.9 (98.6, 99.2)	98.2 (90.1, 100.0)	98.9 (98.6, 99.2)
**Agreement between Xpert and MGIT**	N/N	6524/6621	85/87	6439/6534
	% (95% CI)	98.5 (98.2, 98.8)	97.7 (91.9, 99.7)	98.6 (98.2, 98.8)

Abbreviations: CI, confidence interval; MGIT, Mycobacteria Growth Indicator Tube;

PPV, positive predictive value; NPV, negative predictive value.

a Excluded from the analyses were 249 specimens that were contaminated in MGIT culture and 23 specimens that had a final result of failed on Xpert MTB/RIF testing.

b sensitivity of smear microscopy for detection of MTB, using MGIT as reference standard, was 33/187 (17.6%, [95%CI 12.5, 23.9]) vs. Xpert sensitivity of 62.6% overall (p≤0.001).

c These 54 specimens were culture-negative for *M. tuberculosis*. For 11/54, a non-tuberculous mycobacterium was isolated from culture. For 43/54 the culture was negative for any mycobacterial growth; among these 43 specimens, 32 were smear grade scanty.

Xpert was positive for MTB in 27 specimens that were negative for MTB growth (and not contaminated) by MGIT culture: 25 had no growth in MGIT culture and 2 had growth of a mycobacterium determined not to be MTB by MPB64 antigen testing of the cultured bacteria. Among these 27 specimens, 8 (29.6%) were from participants with current TB treatment, 9 (33.3%) were from participants with prior TB treatment, and 10 (37.0%) were from participants without current or prior TB treatment. Xpert quantification was “low” in 6/27 (22.2%) and “very low” in 21/27 (77.8%). Median months from self-reported TB diagnosis to study sputum collection were 2.5 (range 1–4) and 9 (range 1–43) for participants with current TB treatment and participants with prior TB treatment, respectively. A “false positive” Xpert result was strongly associated with current TB treatment (OR 92.1; 34.9, 243.0); prior TB treatment was also associated with a false positive Xpert test (Table 4).

**Table 4:** Factors associated with “false positive” detection of *M. tuberculosis* by Xpert MTB/RIF.

	False-positive Xpert result for detection of*M. tuberculosis*	Odds Ratio
	N (row %)	95% CI^b^
**No current or prior TB treatment**	10/5764^a^ (0.2)	1
**Current TB treatment**	8/58 (13.8)	92.1 (34.9, 243.0)
**Prior but not current TB treatment**	9/797 (1.1)	6.6 (2.7, 16.2)

Abbreviations: CI, confidence interval.

a four participants who reported not being on current TB treatment but did not know if they had ever been on TB treatment were coded as “no current or prior TB treatment”.

b overall P-value <0.001.

To further explore the impact of current or prior TB on Xpert performance, we calculated Xpert performance stratified by TB history (Table 5). Among participants without current or prior TB treatment, Xpert specificity was exceedingly high at 99.8% (5599/5609), and PPV reached 90.6% (96/106). Specificity and PPV were lowest among participants with current TB treatment (specificity 85.2% [46/54] and PPV 33.3% [4/12]). For Xpert and MGIT culture considered separately, we explored test sensitivity for detection of prevalent TB if participants with “current TB” were considered as prevalent TB cases in addition to participants whose sputum MGIT culture grew MTB. Among the resulting 241 TB cases, MGIT detected 187/241 (77.6%; 95% CI 71.8, 82.7) and Xpert detected 125/241 (51.9%, 95% CI 45.4, 58.3; p≤0.001).

**Table 5:** Performance characteristics of the Xpert MTB/RIF test stratified by participant TB treatment status, using MGIT culture as the reference standard^a^.

		No current or prior TB treatment	Current TB treatment	Prior but not current TB treatment
		N = 5764^b^	N = 58	N = 797
**Xpert sensitivity**	N/N	96/155	4/4	17/28
	% (95% CI)	61.9 (53.8, 69.6)	100 (39.8, 100.0)	60.7 (40.6, 78.5)
**Xpert specificity**	N/N	5599/5609	46/54	760/769
	% (95% CI)	99.8 (99.7, 99.9)	85.2 (72.9, 93.4)	98.8 (97.8, 99.5)
**Xpert PPV**	N/N	96/106	4/12	17/26
	% (95% CI)	90.6 (83.3, 95.4)	33.3 (9.9, 65.1)	65.4 (44.3, 82.8)
**Xpert NPV**	N/N	5599/5658	46/46	760/771
	% (95% CI)	99.0 (98.7, 99.2)	100.0 (92.3, 100.0)	98.6 (97.5, 99.3)
**Agreement between Xpert and MGIT**	N/N	5695/5764	50/58	777/797
	% (95% CI)	98.8 (98.5, 99.1)	86.2 (74.6, 93.9)	97.5 (96.2, 98.5)

Abbreviations: CI, confidence interval; MGIT, Mycobacteria Growth Indicator Tube; PPV, positive predictive value; NPV, negative predictive value.

a Excluded from the analyses were 249 specimens that were contaminated in MGIT culture, 23 specimens that had a final result of failed on Xpert MTB/RIF testing, and 2 specimens from participants for whom data on TB treatment status were missing.

b four participants who reported not being on current TB treatment but did not know if they had ever been on TB treatment were coded as “no current or prior TB treatment”.

Among 144 specimens with MTB detected by Xpert, rifampicin resistance was detected for 7/144 (4.9%), not detected for 134/144 (93.1%), and indeterminate for 3/144 (2.1%). Among the seven specimens that were rifampicin resistant by Xpert, five were rifampicin resistant by MGIT phenotypic testing, and two had no growth in primary MGIT culture so phenotypic susceptibility testing could not be performed.

Costs for equipment and consumables are shown in Table S1. Under the base scenario, the cost per sputum specimen tested was $23.67 for Xpert and $15.55 for the package of smear microscopy plus MGIT culture for MTB detection (Table 6). For the testing scenario of 7000 tested specimens (among which 2.7% of cultures were positive for MTB and subsequently subjected to phenotypic drug susceptibility testing [DST]), the total cost for an Xpert testing strategy was $165,690 and for the package of smear/MGIT culture/MGIT DST was $115,360 (difference of $50,330).

**Table 6:** Diagnostic test costs, per specimen tested and for a testing program including 7000 sputum specimens.

Diagnostic test(s)	Cost per specimen tested ($)	Cost for testing 7000 sputa ($)
Smear + MGIT^a^	15.55	108,850
DST	34.44	–
Smear + MGIT^a^ + DST	–	115,360^d^
Xpert MTB/RIFb,c	23.67	165,690

Abbreviations: MGIT, Mycobacterial Growth Indicator Tube; DST: drug susceptibility testing using the MGIT SIRE system.

a includes the cost of sputum decontamination ($2.35/sputum specimen).

b does not include the cost of sputum decontamination.

c unit cost of Xpert MTB/RIF cartridges was $16.80.

d incorporates the assumption that DST is performed for the 2.7% of specimens with growth of *M. tuberculosis* in culture.

## Discussion

The ‘Thibela TB’ final prevalence survey offered a unique opportunity to evaluate Xpert performance in a population-based TB survey, as the infrastructure was in place for the survey. Moreover, MGIT testing was performed for each specimen, thereby allowing direct comparison between Xpert and MGIT. We reasoned that Xpert performance characteristics, especially PPV, might be different when implemented in a scenario in which relatively few individuals have TB disease (e.g. a prevalence survey, some settings of active/enhanced TB case finding, and passive detection of TB cases in low TB prevalence settings such as the U.S.) versus when applied for passive detection in high TB burden settings. We sought to establish Xpert performance characteristics in this highly structured setting in which MGIT culture was performed in order to inform the potential use of Xpert in future TB prevalence surveys and other initiatives in which TB disease prevalence is relatively low.

We found that the proportion of tests yielding a non-informative result was lower for Xpert than for MGIT, although the overall TB diagnostic yield of Xpert (2.1%) was lower than that for MGIT (2.7%). Xpert sensitivity, using MGIT as the reference comparator, was 62.6% overall, which is lower than that reported for Xpert used for passive case detection in symptomatic pulmonary TB suspects in validation and demonstration studies [13], [14], but similar to that reported by Lawn among consecutive HIV-infected adults enrolling in an ART clinic whose sputum was tested by Xpert regardless of symptoms [22].The lower overall sensitivity in our study was driven by the generally low sputum bacillary burden among TB cases in our prevalence survey – among culture-positive TB cases only 17.6% were smear microscopy positive. However we found that the sensitivity of Xpert in smear positive, culture positive TB cases was 97.0%, which is similar to that reported by others [13], [14]. The exceedingly low observed sensitivity of smear microscopy highlights the inadequacy of this test, at least in the context of a TB prevalence survey in an HIV prevalent setting. NTM were identified in just over 5% of specimens by MGIT culture, whereas, as expected, Xpert was negative in almost all of those. We did not assess the clinical significance of NTM isolates in individual study participants. However, the ability of liquid culture methods to facilitate diagnosis of lung disease caused by NTM and, by contrast, the inability of Xpert to provide such diagnostic information, would make liquid culture advantageous in some clinical circumstances.

As a general principle, the positive predictive value of a test with imperfect specificity is typically adversely affected in scenarios of low disease prevalence since a relatively large proportion of positive tests will be “false positives”. In a recent WHO report, Xpert PPV was calculated to be 74% and 65% under TB prevalence scenarios of 3% and 2%, respectively, when an Xpert specificity estimate of 99% was used [23]. However, in our study in which observed TB prevalence as assessed by MGIT was 2.7%, the Xpert PPV was 81.3%, reflecting a relatively low number of false positive Xpert tests. Furthermore, the demonstrated association of current TB treatment and the results pattern of Xpert positive for MTB but MGIT negative for MTB is compatible with a scenario in which active TB (with MTB organisms) was present and the MGIT was falsely negative, presumably due to antibiotic-mediated suppression of bacterial growth. In a secondary analyses from which participants with current or prior TB were excluded, Xpert PPV was just over 90%, representing only 10 false positives among 5764 tested specimens. Xpert attributes that might contribute to low percentages of falsely positive results include its analytical specificity based on the molecular probe designs, its closed cartridge system that minimizes specimen-to-specimen cross-contamination, and its automated readout that minimizes user interpretation errors. False-positive cultures for MTB are not rare – one literature review identified false-positive cultures in 13 (93%) of 14 studies that assessed >100 patients, and the median false-positive percentage was 3.1% [24].

Molecular tests for TB can detect nucleic acids from non-viable bacilli, and in our study a test result of Xpert positive for MTB but MGIT negative for MTB was strongly associated with current TB. The implications of a positive test result arising from non-viable organisms will vary based on testing circumstances. In our study participants were queried about current TB treatment – those on current TB treatment are arguably a subset of prevalent TB cases, and Xpert detected a higher proportion of them than did MGIT.

A limitation of our study is that Xpert tests were performed on decontaminated sputum pellets rather than on raw sputum. As MGIT culture result was the stated gold standard for TB diagnosis in the context of the ‘Thibela TB’ prevalence survey, every effort was made to ensure that sample handling was optimal for MGIT culture. Therefore raw sputum was not split, a process that can result in nonequivalent fractions. In a large multisite trial of Xpert there was no difference in yield from raw sputum versus decontaminated pellets [13]. Another limitation is that only one respiratory specimen was obtained per participant; testing of additional specimens by culture might have helped to resolve discrepancies in which the Xpert was positive yet the culture was negative. Finally, the relatively low number of TB cases identified in our prevalence survey did not allow us to meaningfully assess Xpert performance characteristics for detection of rifampicin resistance. False-positive rifampicin resistance results from Xpert testing have been reported, and any imperfection in assay specificity would be expected to result in a substantially reduced positive predictive value (for rifampicin resistance) in a setting of low MDR-TB prevalence [14], [16], [17], [18].

We determined the laboratory costs of a testing program using Xpert alone versus a program combining smear microscopy with MGIT culture and MGIT-based DST for MTB-positive cultures. The cost for the Xpert program was 44% greater than that for the microscopy/MGIT program. Xpert cartridge unit costs accounted for just over 70% of Xpert per test cost, and therefore the laboratory costs for an Xpert testing program would be expected to be reduced with declining cartridge costs. Our per test cost results are strikingly similar to those reported by Vassall and sourced from a demonstration study of Xpert use including in South Africa [25]. For the South African study sites, Vassall reported Xpert costs per test of $22.00 and $25.90 assuming Xpert cartridge unit costs of $15.50 and $19.40, respectively; we found an Xpert per test cost of $23.67 given an actual Xpert cartridge unit cost of $16.80. Cost per tested specimen for smear+MGIT culture was $16.82 in the Vassall study versus $15.55 in our study. Our costing analysis incorporated several assumptions that might not apply to future Xpert testing programs. For example, we assumed that a biosafety cabinet was required for Xpert testing, and that Xpert testing was conducted by research staff at a higher pay grade than routine service laboratory staff conducting MGIT testing. While these assumptions applied to our MGIT-based ‘Thibela TB’ prevalence survey laboratory testing program, these aspects are not strictly required for conducting Xpert testing.

In summary, in the context of a MGIT-based TB prevalence survey, Xpert sensitivity was substantially higher than that of smear microscopy but lower than that of MGIT culture. Xpert detected a subset of prevalent TB cases (namely individuals on TB treatment when the prevalence survey sputum was obtain) not detected by MGIT, and the proportion of Xpert tests that were non-informative was lower than that for MGIT. We conclude that Xpert has promise for use as a sole testing strategy in the context of TB prevalence surveys and other initiatives in which prevalence and pre-test probability of TB disease are relatively low. In the context of a TB prevalence survey, Xpert sensitivity less than that of MGIT might be counterbalanced by Xpert ease of use.

## Supporting Information

Table S1 **Unit costs of key equipment and consumables.**
(DOCX)
