# Supplementary material for: Performance Characteristics of the Cepheid Xpert MTB/RIF Test in a Tuberculosis Prevalence Survey
Source: PLoS One. 2012 Aug 15;7(8):e43307. doi: 10.1371/journal.pone.0043307 (PMC3419700; doi:10.1371/journal.pone.0043307)
Supplement: Table S1 — Unit costs of key equipment and consumables. (DOCX) [file pone.0043307.s001.docx]

| **On-Line Supplement/Supporting Information**  Table S1. Unit costs of key equipment and consumables | | | |
| --- | --- | --- | --- |
|  | **Unit (quantity)** | **Unit Cost ($)** | **Source** |
| **CONSUMABLES** |  |  |  |
| Specimen Collection and Transport | per sputum | $2.88 | Lab Records |
| Smoke test | per kit | $93.15 | Lab Records |
| NALC/NAOH/buffer/Water kit | per kit (one per sample) | $2.03 | Lab Records |
| 50ml Conical Centrifuge tubes | per box (500) | $190.32 | Lab Records |
| gloves | per box (100) | $8.60 | Lab Records |
| N95 | per box (100) | $64.95 | Lab Records |
| disposable pipette | per box (500) | $52.92 | Lab Records |
| slides | per box (50 ) | $3.87 | Lab Records |
| ZN stain | per liter | $5.73 | Lab Records |
| 3% Acid ethanol | per liter | $4.87 | Lab Records |
| Methylene Blue | per liter | $3.83 | Lab Records |
| AFB fixative | per 100ml | $10.03 | Lab Records |
| 70% ethanol | per liter | $3.03 | Lab Records |
| Ultraseptin/disinfectant | 6 kg | $460.45 | Lab Records |
| gloves | per box (100) | $8.60 | Lab Records |
| Normal Saline | 9.9 ml | $1.02 | Lab Records |
| N95 masks | per box (100) | $64.95 | Lab Records |
| Pipette Tips: 1000 microliter | per box (800) | $160.22 | Lab Records |
| Pipette Tips: 200 microliter | per box (960) | $70.22 | Lab Records |
| PANTA | one box (100) | $100.32 | Lab Records, FIND website |
| MGIT Growth Supplement | one box (100) | $82.40 | Lab Records, FIND website |
| disposable pipette | per box (500) | $52.92 | Lab Records |
| Blood Agar plate | per packet (10) | $4.92 | Lab Records |
| Capillia Test | per test | $1.99 | Manufacturer |
| MGIT Tube | per box (100) | $300.95 | Lab Records, FIND website |
| SIRE kit | per kit (35) | $126.40 | Lab Records, FIND website |
| Taq | per kit (96) | $251.22 | Lab Records |
| Xpert kit | per kit (10) | $16.80 | Lab Records, FIND website |
| Xpert freight | per kit (10) | $1.61 | Lab Records, FIND website |
| **EQUIPMENT** |  |  |  |
| Multifuge plus accessories | per instrument | $26,408.55 | Lab Records |
| Vortex | per instrument | $358.27 | Lab Records |
| Shaker | per instrument | $4,172.75 | Lab Records |
| Biosafety Cabinet | per instrument | $3,754.72 | Lab Records |
| Biosafety Cabinet Filter replacement | yearly | $1,893.12 | Lab Records |
| Biosafety Cabinet Decontamination | q 6months | $184.15 | Lab Records |
| Light Bulb for microscope | per bulb | $7.88 | Lab Records |
| Light Microscope | per instrument | $4,012.67 | Lab Records |
| Bactec MGIT 960 | per instrument | $8,254.64 | Lab Records |
| Epicenter software | -- | $26,943.96 | Lab Records |
| Barcode scanner | per instrument | $186.30 | Lab Records |
| Thermometer #1 | per instrument | $7.85 | Lab Records |
| Thermometer #2 | per instrument | $34.97 | Lab Records |
| thermocycler | per instrument | $6,748.89 | Lab Records |
| Ultrasonicator | per instrument | $1,878.79 | Lab Records |
| GTBLOT maintanence | semi-annual | $573.24 | Lab Records |
| GT BLOT | per instrument | $33,677.80 | Lab Records |
| microfuge | per instrument | $8,438.80 | Lab Records |
| microfuge rotor | per instrument | $595.74 | Lab Records |
| UPS (power supply) | per instrument | $161.51 | Lab Records, FIND website |
| Xpert Instrument | per instrument | $15,991.62 | Lab Records, FIND website |
| Xpert Computer/Software/connectors | per instrument | $14,186.68 | Lab Records, FIND website |
| Xpert calibration | per 1800 runs | $2,082.00 | Lab Records, FIND website |
